# Arthroscopic-Assisted Treatment of a Reversed Hill-Sachs Lesion: Description of a New Technique Using Cerament

**DOI:** 10.1155/2015/789203

**Published:** 2015-01-26

**Authors:** S. Bark, F. Renken, A. P. Schulz, A. Paech, J. Gille

**Affiliations:** Department of Trauma and Reconstructive Surgery, University of Schleswig-Holstein, Campus Lübeck, Germany

## Abstract

*Purpose*. Impaction fractures of the anterior aspect of the humeral head, the reversed Hill-Sachs lesion, are common in posterior shoulder dislocation. We present a new technique to address these lesions arthroscopic-assisted with the use of a bone substitute. *Methods*. We report the case of a 45-year-old male with a reversed Hill-Sachs lesion after posterior shoulder dislocation. Initially a glenohumeral arthroscopy is performed to address concomitant intra-articular injuries. Guided by the k-wire a cannulated sizer was inserted for reduction of the fracture under arthroscopic visualization. For reduction of the impacted part of the humeral head the subcortical defect was filled with an injectable bone substitute (Cerament) to prevent secondary dislocation. *Results*. X-ray at follow-up 6 months after the index procedure documents the bony remodeling of the bone substitute. At that time the patient was pain-free (VAS 0) and satisfied with the outcome (Constant score: 78, Rand-36 score: 84, Rowe score: 81) with a good ROM. *Conclusions*. In conclusion, arthroscopic-assisted reconstruction of reversed Hill-Sachs lesions with an injectable bone substitute is feasible and may provide patients with all the benefits of an anatomic reconstruction with decreased risks related to open surgery.

## 1. Introduction

Traumatic posterior dislocation of the glenohumeral joint represents a rare injury. Often it may go undetected due to the lack of a correct X-ray projection or missing clear presentation of clinical signs, which usually consist of pain and unability to do complete elevation and external rotation [1, 2]. Posterior dislocation of the shoulder may result in an impression fracture of the anterior aspect of the humeral head, the so-called reverse Hill-Sachs lesion which has been reported in up to 86% of patients following a first time posterior dislocation [3]. These lesions may cause significant clinical symptoms and are able to increase the risk of recurrent instability. The causes of posterior shoulder dislocations are usually severe traumatic events or sudden violent internal rotatory muscles contraction (e.g., during a convulsive seizure) [1]. The treatment depends on the size of the defect and various surgical techniques have been described. It varies from conservative treatment to surgical options such as bone grafting, subscapularis tendon transfer, and arthroplasty [4]. Most of these techniques are invasive and necessitate an extensive surgical approach to the shoulder. We present the case of a 45-year-old man with a reversed Hill-Sachs lesion due to a convulsive seizure with a posterior shoulder dislocation. We describe a minimal invasive, arthroscopic-assisted technique to address the reverse Hill-Sachs lesion with bone cement augmentation (Cerament, Bonesupport GmbH, Frankfurt/Germany).

## 2. Case Report

**Figure 1:**
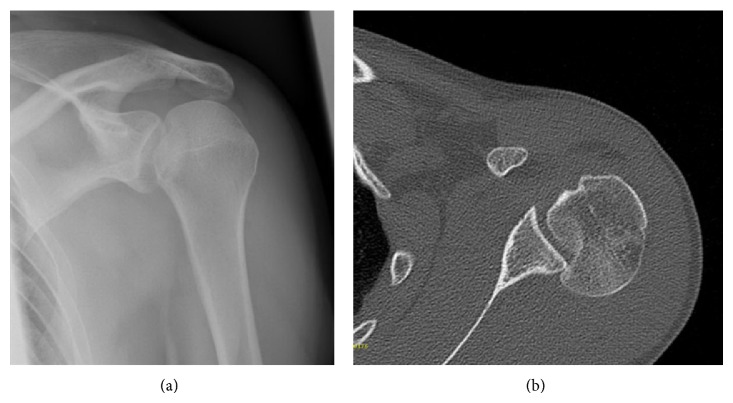
X-rays (a) and CT (b) showing the joint fixed in dorsal dislocation with the presence of a reversed Hill-Sachs defect.

A 45-year-old man sustained a posterior dislocation of his left shoulder following a convulsive seizure while he was playing soccer. He did not have a history of any previous injury or symptoms regarding his shoulder. X-rays and CT showed the joint fixed in dorsal dislocation with the presence of a reversed Hill-Sachs defect affecting 30% of the articular surface (Figure 1). Behind the background of the literature, the decision for a surgical approach was made [4].

### 2.1. Surgical Technique

**Figure 2:**
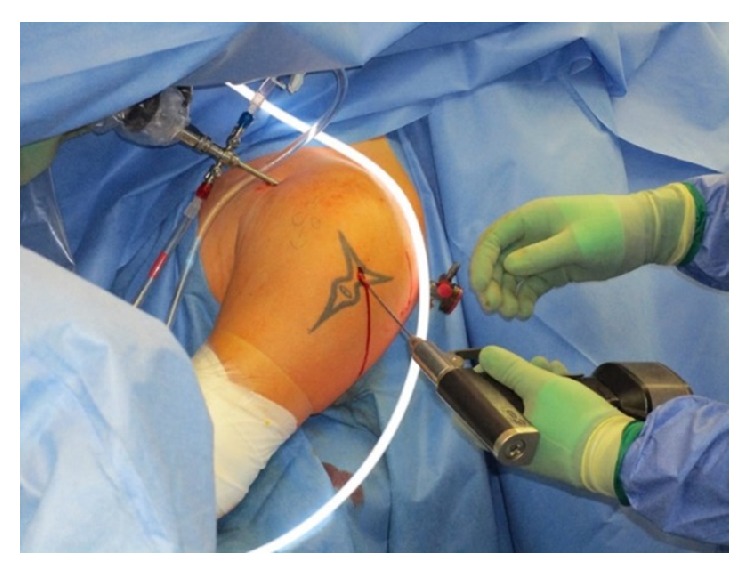
Intraoperative setting with a lateral view (left shoulder). The arthroscope is placed in the anterior portal. A drill pin is placed from a lateral, minimal invasive approach in the area of the reversed Hill-Sachs lesion under fluoroscopic visualization and simultaneous visual control by arthroscopy.

**Figure 3:**
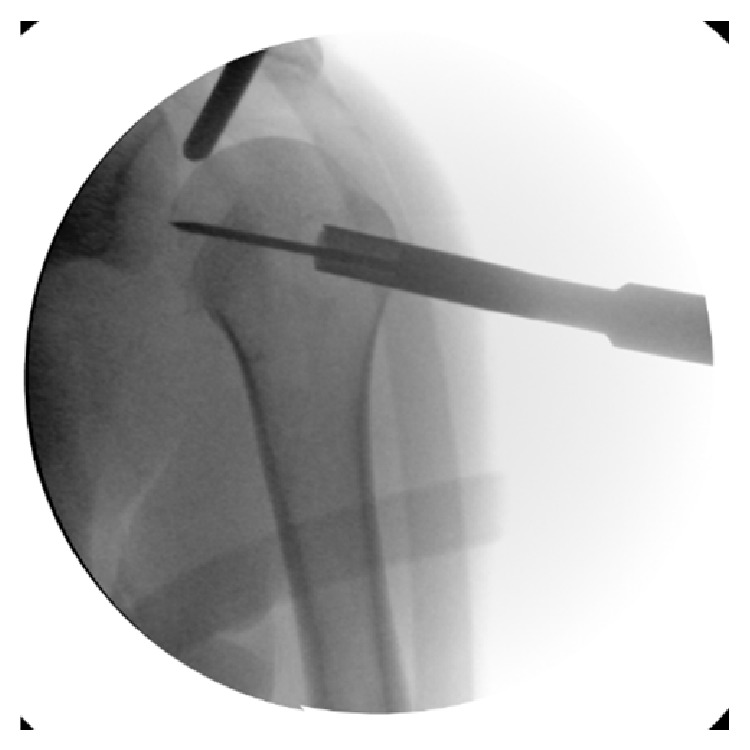
Fluoroscopic visualization during the index procedure. Guided by the k-wire a cannulated sizer (8 mm diameter, BioMatrix CRD instruments, Arthrex) was inserted for reduction of the fracture under arthroscopic visualization.

**Figure 4:**
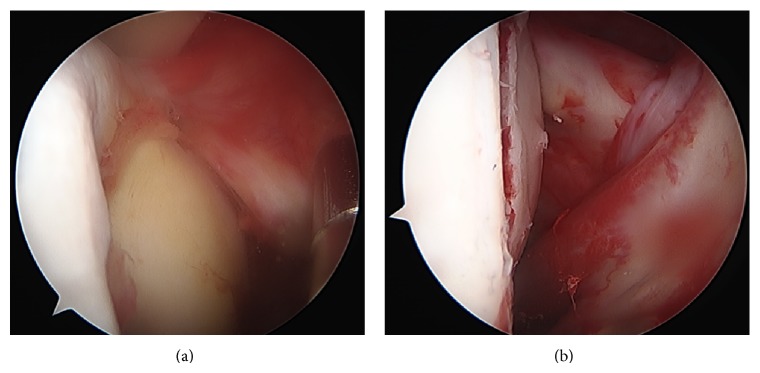
Arthroscopic view (posterolateral portal) of the humeral head before (a) and after (b) reduction of the reversed Hill-Sachs lesion. For internal fixation the subcortical defect was filled with an injectable bone substitute (Cerament).

The patient was placed in beach chair position with an image intensifier perpendicular to the patient's axis from the contralateral side to allow arthroscopy and radiographs simultaneously. Under general anaesthesia, a closed reduction of the glenohumeral joint was performed and standard arthroscopic portals were placed. At the anteromedial humeral head the reversed Hill-Sachs lesion was visualized (Figure 4(a)); the findings were in accordance with the preoperative X-rays and CT scans (Figure 1). No more injuries were found in the anterior shoulder region with an intact tendon of the subscapularis, the biceps brachii tendon, and an intact labrum. Through a lateral approach a k-wire was placed into the central defect of the Hill-Sachs lesion (Figure 2); it can be helpful to guide the k-wire by a tibial guide used in cruciate ligament surgery (Arthrex, USA). Guided by the k-wire a cannulated sizer (8 mm diameter, BioMatrix CRD instruments, Arthrex) was inserted for reduction of the fracture under arthroscopic visualization (Figure 3). For internal fixation the subcortical defect was filled with an injectable bone substitute (Cerament) to prevent secondary dislocation. After hardening of the bone substitute the reconstruction of the humeral head was documented by arthroscopy (Figure 4(b)). Wounds were closed and the portals were covered with sterile dressings.

**Figure 5:**
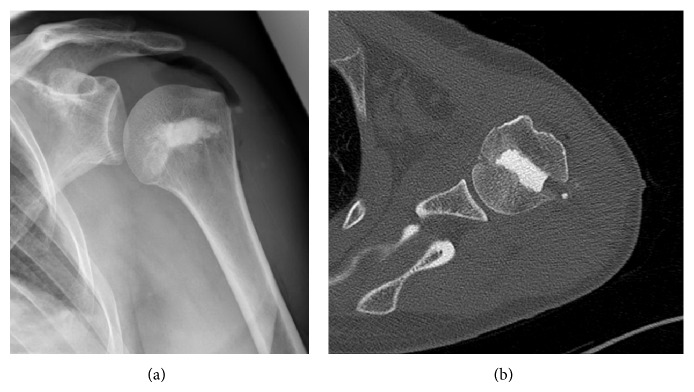
X-ray (a) and CT (b) two days after index procedure showing an intact glenohumeral articulation and a restored humeral head.

X-ray and CT scan of the shoulder two days after the index procedure showed an intact glenohumeral articulation with a restored humeral head and a subcortical defect filled up with Cerament (Figures 5(a) and 5(b)).

The shoulder was braced in neutral rotation for 2 weeks postoperatively and easy functional physiotherapy was started.

### 2.2. Results

**Figure 6:**
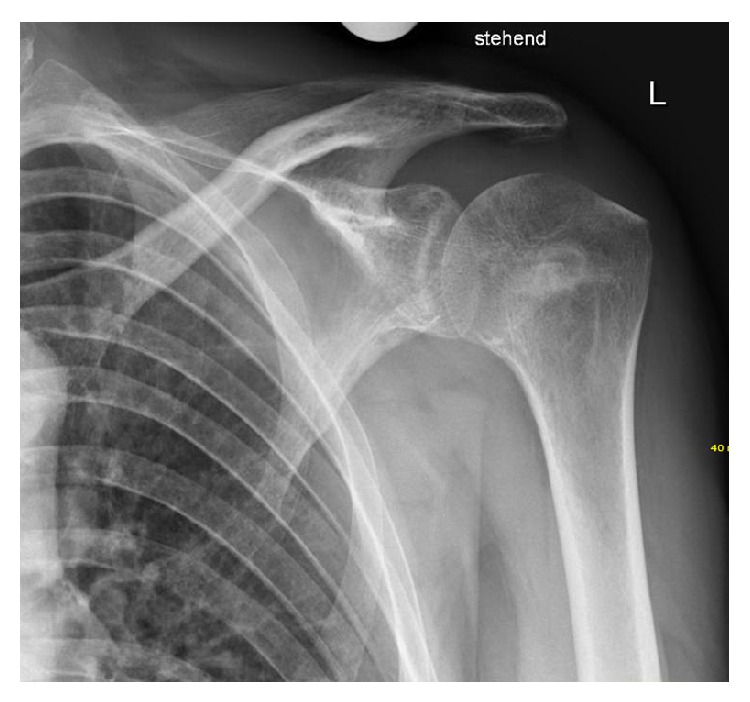
X-ray (a.p. view) at 6-month follow-up documenting the bony remodeling of the bone substitute.

**Figure 7:**
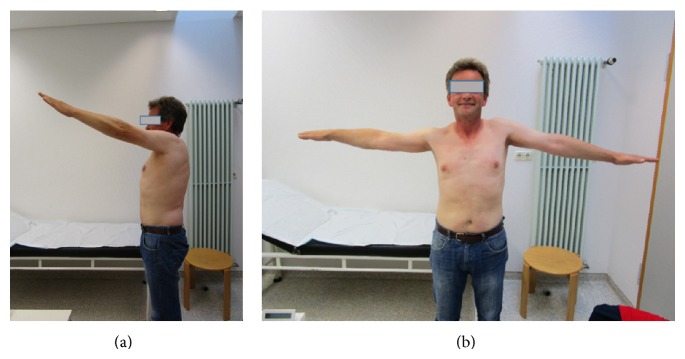
(a), (b) At follow-up 6 months after the index procedure the patient shows a good functional outcome and is very satisfied with the result.

X-ray at follow-up 6 month after the index procedure documents the bony remodeling of the bone substitute (Figure 6). At that time the patient was pain-free (VAS 0) and satisfied with the outcome (Constant score: 78, Rand-36 score: 84, Rowe score: 81) with a good ROM with 90° abduction and 110° elevation (Figures 7(a) and 7(b)).

## 3. Discussion

Posterior shoulder dislocation is an uncommon injury with a reported prevalence of 1.1 per 100,000 per year; many of these injuries are missed at the time of initial presentation [3, 5]. Regarding the treatment of the reversed Hill-Sachs lesion, there is consent in literature that early treatment and anatomic reconstruction of the defect result in better outcome than delayed surgery or nonanatomic procedures [4]. Many different procedures have been described in the past to treat the defect due to the humeral impaction fracture: shift of the subscapularis tendon into the defect, shift of the tuberosity and the tendon, autologous bone grafting and capsular repair, rotation osteotomy of the proximal humerus, defect filling using screws and osteoconductive material, allograft reconstruction, and shoulder arthroplasty [2, 6–9]. Most of these techniques are open surgical procedures. The presented case shows an arthroscopic-assisted, minimal invasive approach to address reversed Hill-Sachs lesions. This approach enables one to use smaller incisions with decreased associated risks. Compared to open surgery, the described arthroscopic technique may offer following advantages:

- minimal soft tissue trauma,
- minimal blood loss,
- complete shoulder inspection and treatment of concomitant intra-articular injuries.

The arthroscopic-assisted approach provides surgeons with an opportunity to treat concomitant intra-articular pathologies, which otherwise might have been missed. In a former series, 86% of posterior shoulder dislocations were associated with traumatic intra-articular lesions [10]. We are not aware of any study that reported on revisions after failed reversed Hill-Sachs-lesion repair due to overlooked intra-articular pathology. However, persistent shoulder pain after an otherwise successful index procedure may be related to concomitant injuries [11].

Arthroscopy provides direct visualization of the humeral head impression and may allow a higher accuracy in reduction of the articular surface leading to better clinical outcomes compared to open procedures. Besides this, the need to use fluoroscopy and the X-ray time are reduced; it has been our experience that single-shot-fluoroscopy at the end of surgery is enough.

As described by McLaughlin, the extent of humeral head lesions associated with posterior shoulder dislocation may influence the choice of treatment [6]. Lesions involving less than 20% of the articular surface seem to do well with nonoperative treatment. The first therapeutic option for osteochondral impressions between 25 and 40% is still open to debate; it is the surgeon's choice to perform open reduction and osteosynthesis or prosthesis. Total- or hemiarthroplasty is considered necessary for lesions superior to 40% [2, 6].

In the presented case, the defect was elevated arthroscopically assisted; elevation of the defect has proven to be clinically and radiographically successful in restoring the normal anatomy of the joint surface in patients suffering from a Hill-Sachs lesion [12]. Reconstruction of the humeral head by elevation of depressed cartilage and subchondral relining with cancellous bone graft has been described by several authors. The advantage of our technique using Cerament is twofold: on the one hand there is no donor site morbidity and on the other site it can be performed arthroscopically.

There are limitations that need to be acknowledged and addressed regarding the present study. One limitation has to do with the extent to which the findings can be generalized beyond the case studied. Another limitation is the lack of a follow-up group. However, these limitations can be seen as fruitful avenues for future research under the same theme.
